# A new large-scale gravitational complex discovered in the Gulf of Squillace (central Mediterranean): tectonic implications

**DOI:** 10.1038/s41598-023-40947-3

**Published:** 2023-09-07

**Authors:** Giacomo Mangano, Silvia Ceramicola, Tiago M. Alves, Massimo Zecchin, Dario Civile, Anna Del Ben, Salvatore Critelli

**Affiliations:** 1 Department of Environmental Engineering, University of Calabria, 87036 Arcavacata di Rende, CS Italy; 2 National Institute of Oceanography and Applied Geophysics – OGS, 34010 Sgonico, TS Italy; 3 3D Seismic Laboratory – School of Earth and Environmental Sciences, Cardiff University, Cardiff, CF10 3AT UK; 4 Department of Mathematics and Geosciences, University of Trieste, 341227 Trieste, Italy

**Keywords:** Environmental sciences, Natural hazards

## Abstract

Seismic reflection (2D/3D), borehole and bathymetric data are used to recognize a new gravitational complex in the Gulf of Squillace, Southern Italy, named the *Squillace Complex*. The complex has a NE-striking headwall connected to a basal detachment formed between Messinian evaporites and Tortonian shales. Its sense of movement changes to a W–E direction in the toe region. In plan view, the Squillace Complex is marked by the presence of sinuous and continuous seafloor scarps, just a few kilometers offshore, over an elongated morphological high. Seismic-well ties reveal that the complex was initiated in the Zanclean (~ 4 Ma) and continued its movement into the Gelasian (~ 2.1 Ma) at an average rate of 1.9 mm/year. Movement slowed down in the Calabrian (middle Pleistocene) and continued until the present day at a lower rate of 0.1 mm/year. Gravitational collapse of the *Squillace Complex* correlates with discrete contractional/transpressional events affecting the Calabrian region, which caused basin shortening and the temporary arrest of Calabrian Arc migration. These episodes resulted in tectonic uplift in the study area after 0.45 Ma (Late Pleistocene). Conversely, the complex’s slower movement recorded since the Calabrian (middle Pleistocene) is associated with slab rollback of the Ionian plate under the Calabrian Arc.

## Introduction

Sediment remobilization on continental margins varies in scale and importance, from discrete instability events (mass transport deposits or MTDs), to recurrent mass-wasting capable of producing stacked successions of chaotic, disturbed sediment, which are named mass transport complexes (MTCs)^1^. Gravitational complexes are an order of magnitude larger than MTCs, and form kilometer-scale features resulting from gravity-induced deformation of strata on continental slopes. They may involve the remobilization of up to 4 km of strata over a basal detachment, usually an overpressured shale interval as offshore Nigeria and Namibia, or an evaporitic unit as in SE Brazil and West Africa^2–4^. Overlapping with the largest of MTCs in nature, gravitational complexes reveal an up-dip extensional headwall domain and a downdip contractional toe region, both linked via a basal detachment that accommodates the bulk of movement in the complex per se^5–9^. They reflect large-scale collapse in diverse geological environments such as passive, convergent and strike-slip continental margins, as well as volcanic regions. Gravitational complexes are thus associated with the generation of a significant gravitational potential along a continental margin, or basin shoulders. This gravitational potential is enhanced during phases of active tectonism and uplift via gradual slope oversteepening, as well as during episodes of high sediment supply and related delta front propagation^10–13^.

**Figure 1:**
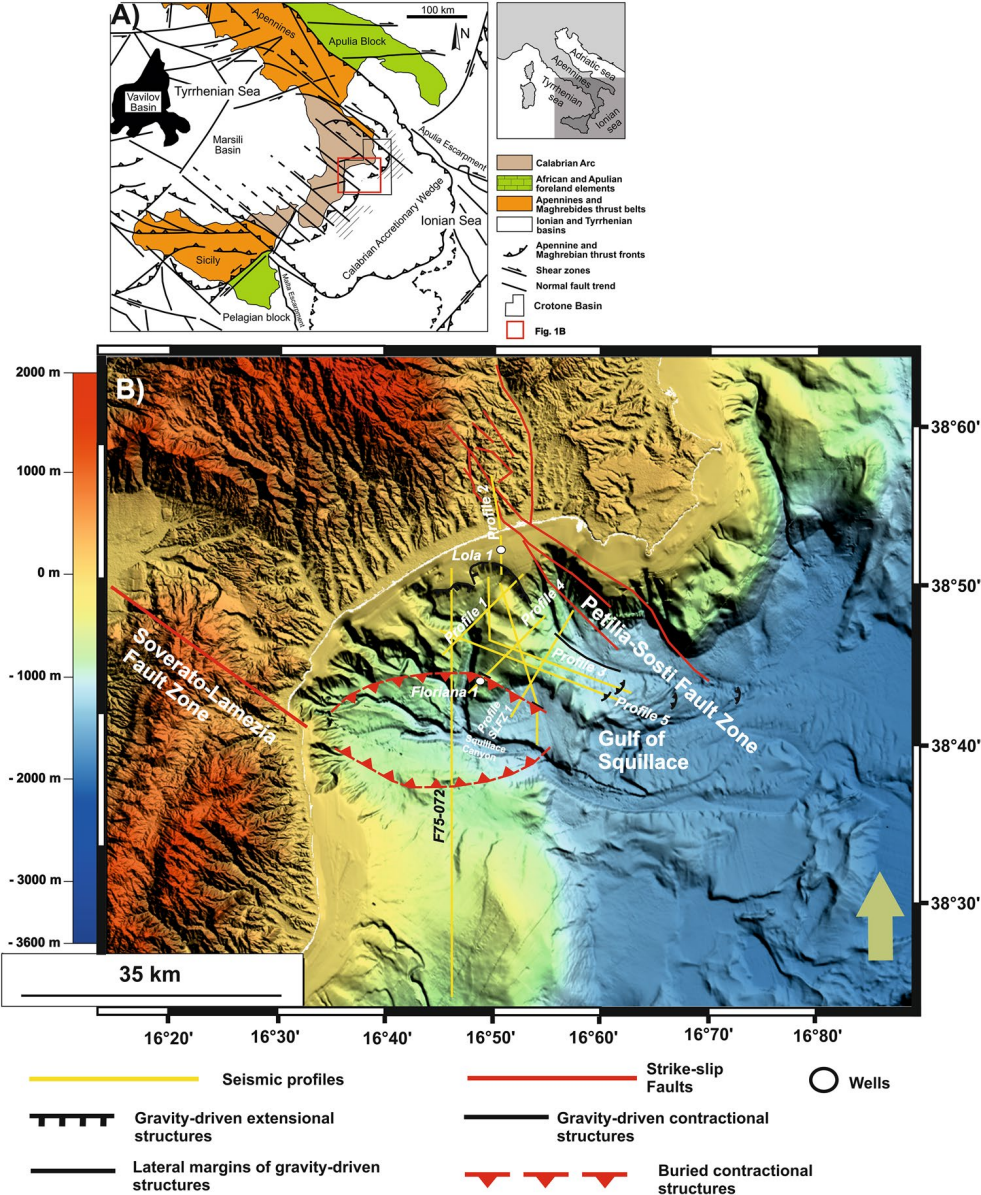
(**A**) Simplified structural map of the Calabrian Arc, which is located between the Neogene mountain chains of the Southern Apennines to the north and the Maghrebides of Sicily to the southwest (modified from Civile et al.^52^). (**B**) Digital Terrain Model (DTM) in a UTM 33 (WGS84) projection highlighting the onshore and offshore parts of the Gulf of Squillace. This gulf is bounded by the two NW-trending Soverato-Lamezia and Petilia-Sosti fault zones, and the eastern sector of the Sila Massif. The locations of seismic profiles and wells are also shown. The DTM map was compiled using: (i) land data derived from SRTM (Shuttle Radar Topography Mission), worldwide digital elevation data with a 30 m (1 arc-second) resolution released by NASA (SRTM PLUS Version 3.0) and made available by the U.S. Geological Survey (https://lpdaac.usgs.gov/search/), and (ii) the TinItaly DEM, a digital elevation model of the whole Italian territory that is available as a 10 m cell size grid (http://tinitaly.pi.ingv.it/Download_Area2.html)^53,54^. The multibeam bathymetry data (MBES) shown were acquired and processed by OGS in 2009 during the MAGIC (MArine Geohazards along the Italian Coasts) Project funded by the Italian Civil Protection.

**Figure 2:**
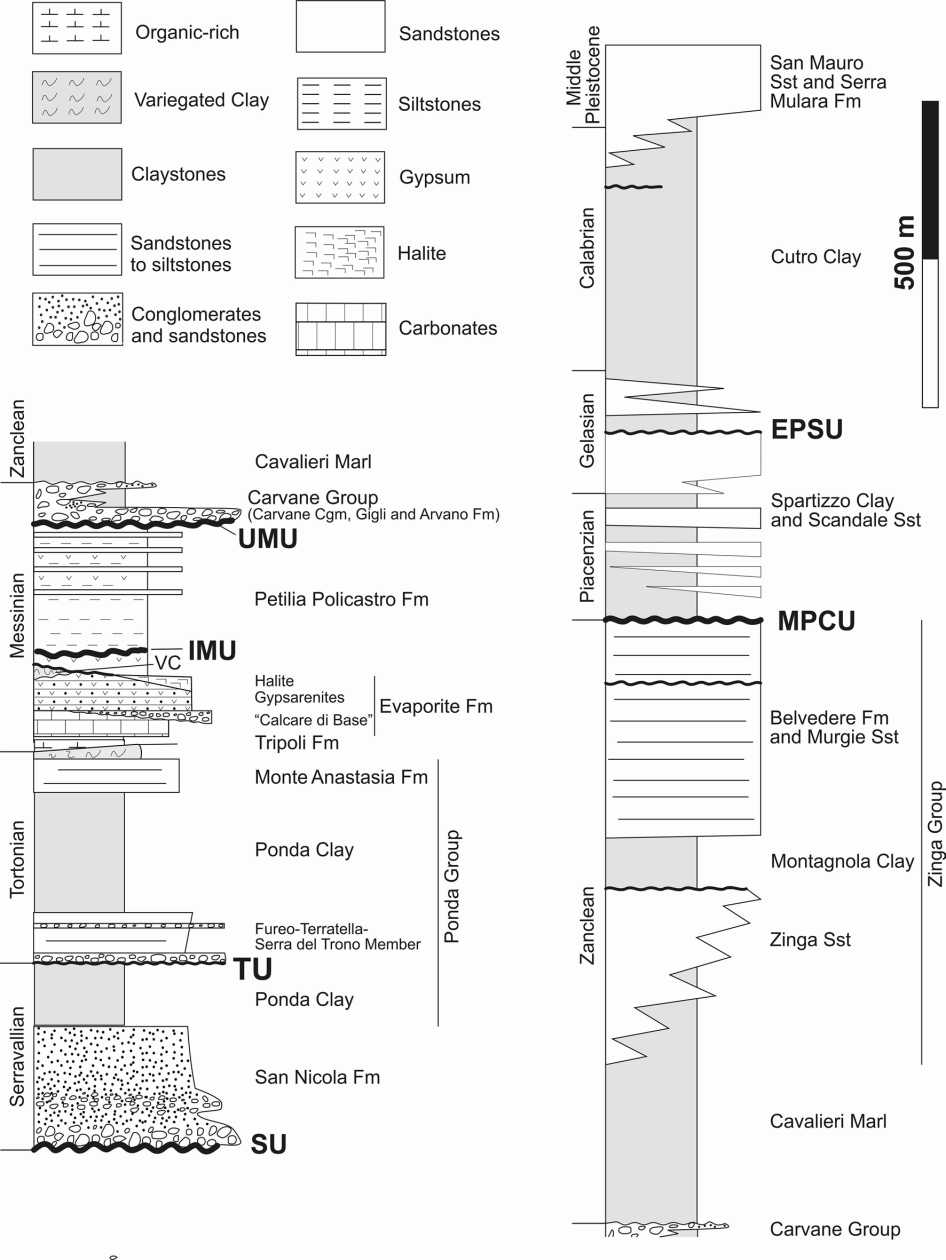
Simplified stratigraphy of the Crotone Basin and its onshore sector (modified from Zecchin et al.^19^). Formations and unconformities are shown in the figure. *SU* Basal Unconformity, *EPSU* Early Pleistocene Unconformity, *IMU* Intra-Messinian Unconformity, *MPCU* Mid-Pliocene Unconformity, *TU* Tortonian Unconformity, *UMU* Upper Messinian Unconformity.

Located in the central Mediterranean region, the study area is part of the Gulf of Squillace and its Neogene to Quaternary forearc depocenter—the Crotone Basin—located on the Ionian sector of the Calabrian Arc, Southern Italy^14–24^. The stratigraphy of the Crotone Basin is closely related to Calabrian Arc kinematics, which has been dominated by alternating phases of forward migration and associated basin subsidence, and collision between the Arc itself and adjacent tectonic plates. Episodes of tectonic collision are associated with the formation of regional stratigraphic unconformities^19^ (Figs. 1, 2 and Table 1). The Crotone Basin is also known by its ~ 1500 km^2^ landslide, the so-called *Crotone Mega-landslide*. This mega-landslide has been controlled, since the Pliocene (Zanclean), by the development of a set of NW-trending strike-slip faults crossing the Calabrian Arc^14,18,20,21^. The Petilia-Sosti and the Lamezia-Soverato Fault Zones are two of such NW-striking faults and cross the SW and NE boundaries of the Gulf of Squillace, whose development has been itself controlled by a E-W-oriented flower structure since the Messinian^25^. In seismic data, all these fault zones show signs of neotectonic activity and nearby gravity-driven instability^18,20–26^. However, while the Crotone mega-landslide has been largely documented in the literature, there is still scarce information about similar large-scale deposits in the Gulf of Squillace. In the published literature, the recognition of potential areas of gravitational collapse has been only attained by Ceramicola et al.^27^ based on bathymetric and sub-seabed data, which allowed for the recognition of fault-bounded sediment blocks and slope-parallel sediment undulations deforming the seabed and strata below. Following the pioneer work of Ceramicola et al.^27^, the aims of this work are:

1. To describe the full extension and geometry of slope instability features in the Gulf of Squillace, south of the city of Crotone;
2. To estimate the ages of any slope instability features recognized in seismic and borehole data;
3. To clarify the mechanisms triggering the interpreted slope instability features, correlating them with the geodynamic evolution of the central Mediterranean region.

**Table 1:** Illustration of the main geological events occurred during the evolution of the Gulf of Squillace, and constituting Crotone Basin.

	Main geological event
Stage	
Gelasian–Holocene	Marsili sub-basin oceanization in the Tyrrhenian at 2.1 Ma
Piacenzian	Vavilov sub-basin spreading phase in the Tyrrhenian
Zanclean	Vavilov sub-basin first spreading in the Tyrrhenian area
Messinian	Messinian Salinity Crisis
Tortonian	Subsiding and transgressive conditions
Serravallian	Crotone Basin opening
Unconformity (Age in Ma)	
MPSU—Mid-Pleistocene unconformity (1.1)	Differential motion between the Calabrian Arc and the southern Appennines
EPSU—Early Pleistocene unconformity (2.4)	Final collision between the NE part of the Calabrian Arc and the Apulian margin
MPCU—Mid-Pliocene unconformity (3.6)	Convergence between the Calabrian Arc and the continental crust of the Apulian margin
ZS—Zanclean unconformity	Transgressive conditions
UMU—Upper Messinian unconformity (5.33)	Collision and temporary coupling of the NE part of the Calabrian Arc with the Apulian margin
IMU—Intra-Messinian unconformity (5.6)	Sea-level drop
TU—Tortonian unconformity (11.63)	Basin shoulder tilting
SU—Serravallian unconformity (13.82)	Basin shoulder tilting

Also described are the main geological events that led to the formation of main stratigraphic unconformities.

These aims are accomplished by integrating offshore 3D seismic reflection data, borehole and morpho-bathymetric data. These new results not only provide new information on potential geohazards affecting Southern Italy, but will be also helpful in the recognition of similar slope instability features worldwide.

## Structural analysis of gravitational collapse features in the Gulf of Squillace

Detailed interpretation of 3D data in the Gulf of Squillace allowed us to divide a geobody, named in this work as *Squillace Complex*, into three structural domains based on the recognition of across-strike variations in structural style; the headwall, translational and toe domains also observed on mass-transport deposits^26,28–29^. These three domains are linked via a basal detachment consisting of a high-amplitude reflector with a listric shape, which displaces Plio-Pleistocene strata and propagates from the base of the Messinian Unit.

### Headwall domain

**Figure 3:**
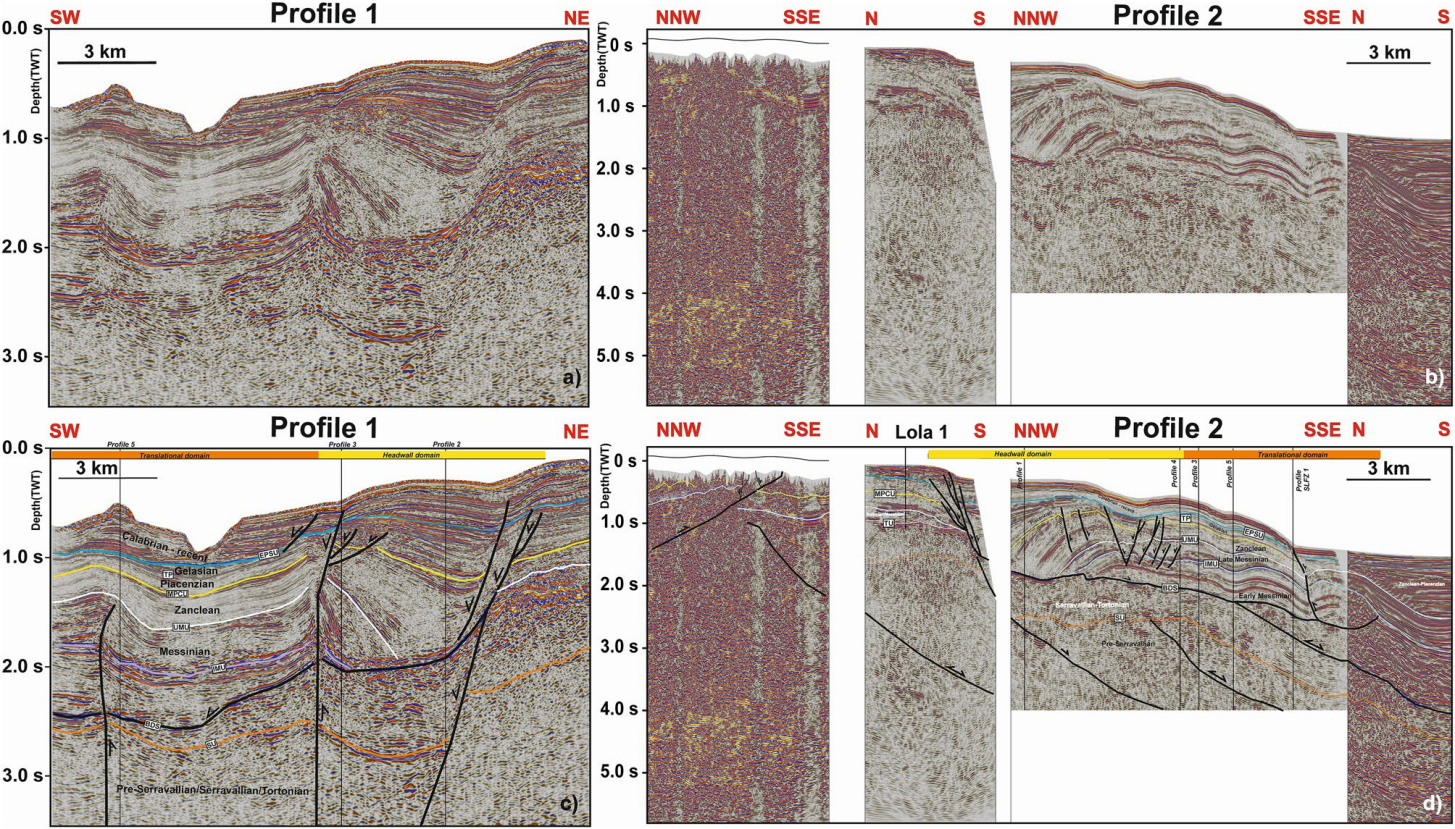
(**a**) Uninterpreted seismic Profile 1; (**b**) Uninterpreted seismic Profile 2; (**c**) the SW-NE-oriented seismic Profile 1 documenting the headwall region and the translational domain of the MTC recognized in the Gulf of Squillace. Black-colored segments indicate fault lineaments. (**d**) Seismic Profile 2 is composed of four sections with different orientations. The headwall region and the translational domain, as also shown in Profile 1, are imaged and interpreted in this figure. The profiles also highlight the presence of rotated blocks and deformed, gently folded strata above a prominent basal detachment.

**Figure 4:**
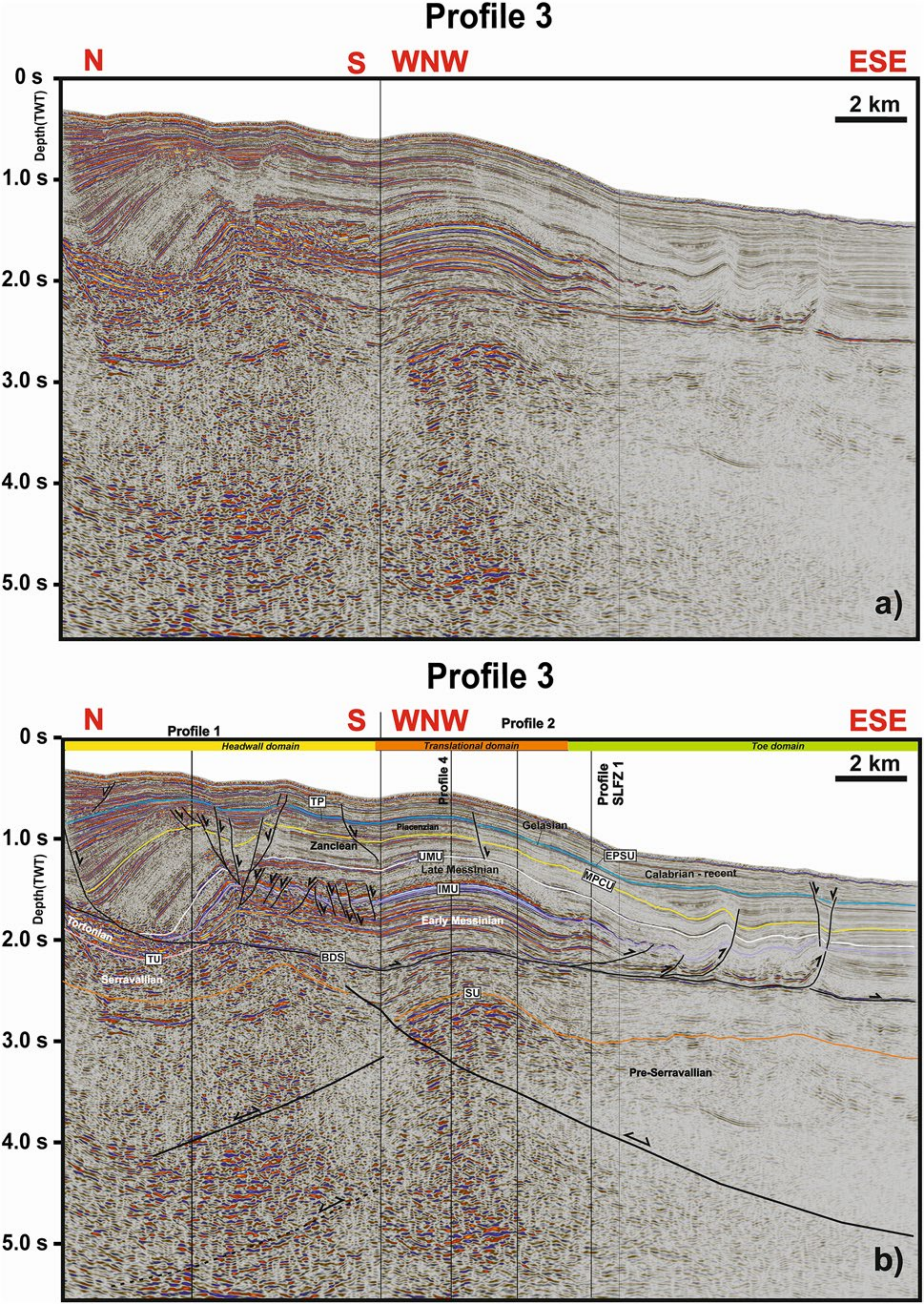
(**a**) Uninterpreted, and; (**b**) Interpreted seismic Profile 3 showing the headwall domain, the translational domain and the toe domain of the *Squillace Complex*. The profile highlights the changes in faulting and slope deformation styles from the complex’s headwall to the translational domains. Seismic data were provided by ENI Natural Resources (see Fig. 1 for their location on map).

**Figure 5:**
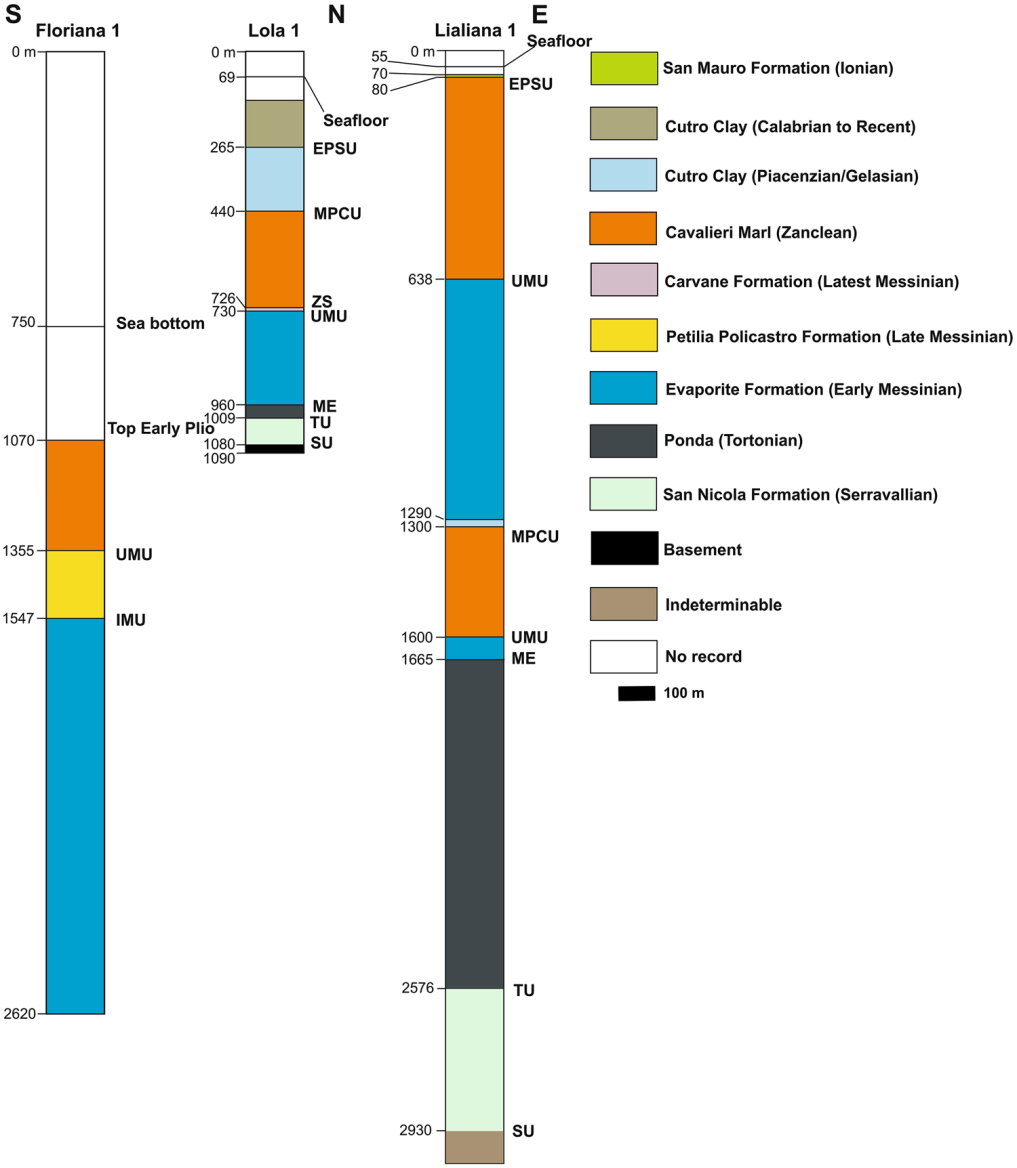
Summary of lithological data from wells Floriana 1 and Lola 1 located in the Gulf of Squillace. Well Floriana 1 and its location are available at http://www.videpi.com/videpi/videpi.asp. Well Lola 1 was provided by ENI National Resources.

The headwall domain of the Squillace Complex occurs 1.5 to ~ 10 km away from the coastline, along its strike, and forms a ~ 1.3 s two-way time (TWT) tall bathymetric feature. It essentially comprises a steep headwall fault (~ 60°) dipping to the SSW (Figs. 3 and 4). Based on stratigraphic data from wells Lola 1 and Floriana 1 (Fig. 5), one can verify that this headwall fault is rooted in the Messinian-age Evaporite Formation, propagating through the Cutro Clay up until it reaches the seafloor (Fig. 3c). Undeformed Tortonian-Quaternary strata are juxtaposed against the headwall fault, downslope from which rotated tilt blocks are observed (Figs. 3, 4). Here, tilt blocks with a thickness of approximately 1.0 s TWT (1000 m) show characteristic syntectonic growth within Zanclean (Cavalieri Marl) and Piacenzian-Gelasian strata, the latter of which are interpreted to be part of the Cutro Clay based on data from Lola 1 and Floriana 1 (Figs. 3c,d, 5). While the Calabrian (middle Pleistocene) to recent strata show nearly parallel reflector in Profiles 1 and 2 (Fig. 3), this same interval is marked by a fan-like geometry in Profile 3 (Fig. 4). Strata with a fan geometry are characterized by their low- to medium-amplitude internal reflections.

**Figure 6:**
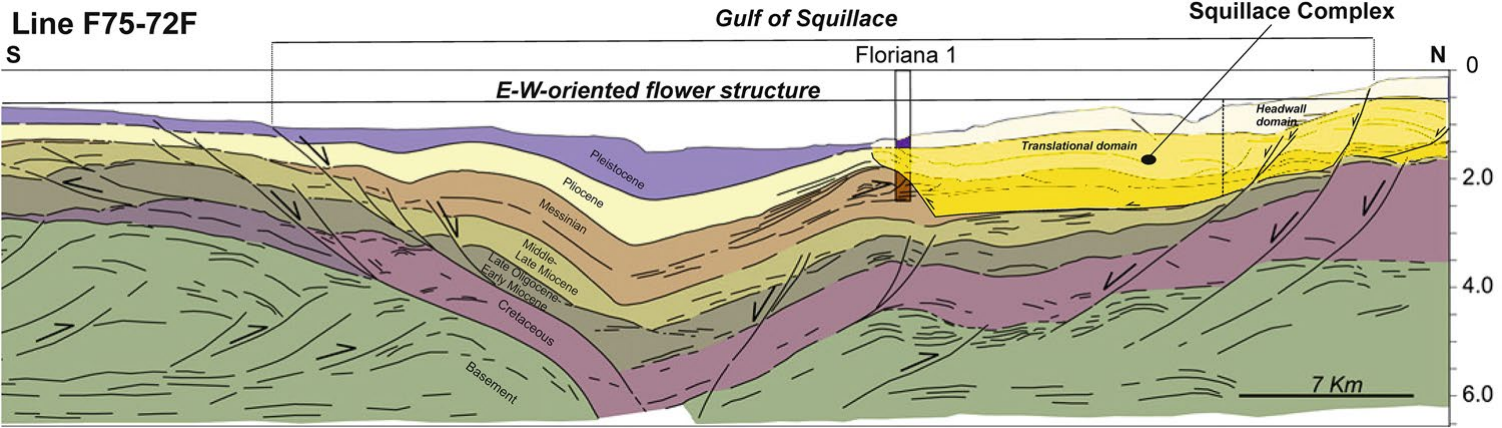
Interpreted seismic line F75-072F (modified from Capozzi et al.^30^). A gravitational complex can be identified in the Gulf of Squillace by the recognition of a south-dipping listric fault with associated growth strata linked via a sub-horizontal basal shear surface, to a N-dipping compressional structure. Note the presence of syntectonic growth strata in the upper part of the Messinian unit interpreted in this seismic profile.

The available 3D seismic data do not permit a complete definition of the headwall domain of the *Squillace Complex* to the W, where its remains unconstrained. Nevertheless, the presence of a headwall to the west of the interpreted 3D seismic volume is supported by the line drawing F75-072F from Capozzi et al.^30^, where a series of S-dipping listric faults, with associated growth strata, is shown to sole out within a sub-horizontal basal detachment (Fig. 6). One of the headwalls, developing for ~ 25 km, coincides with an arcuate NE-trending shelf edge that was sculpted by slope instability processes^27^. In fact, the swath morphobathymetric map in Fig. 1 documents the presence on the seafloor of multiple branches of the Squillace Canyon cutting through the continental shelf. It should be noted the presence of narrow vertical zones of disruption and dimmed amplitudes at the top of the Messinian and Piacenzian successions near the headwall domain. The downslope displacement of the *Squillace Complex* reaches 3.6 km in the strata overlying the Messinian unconformity (Figs. 2 and 3d).

### Translational domain

**Figure 7:**
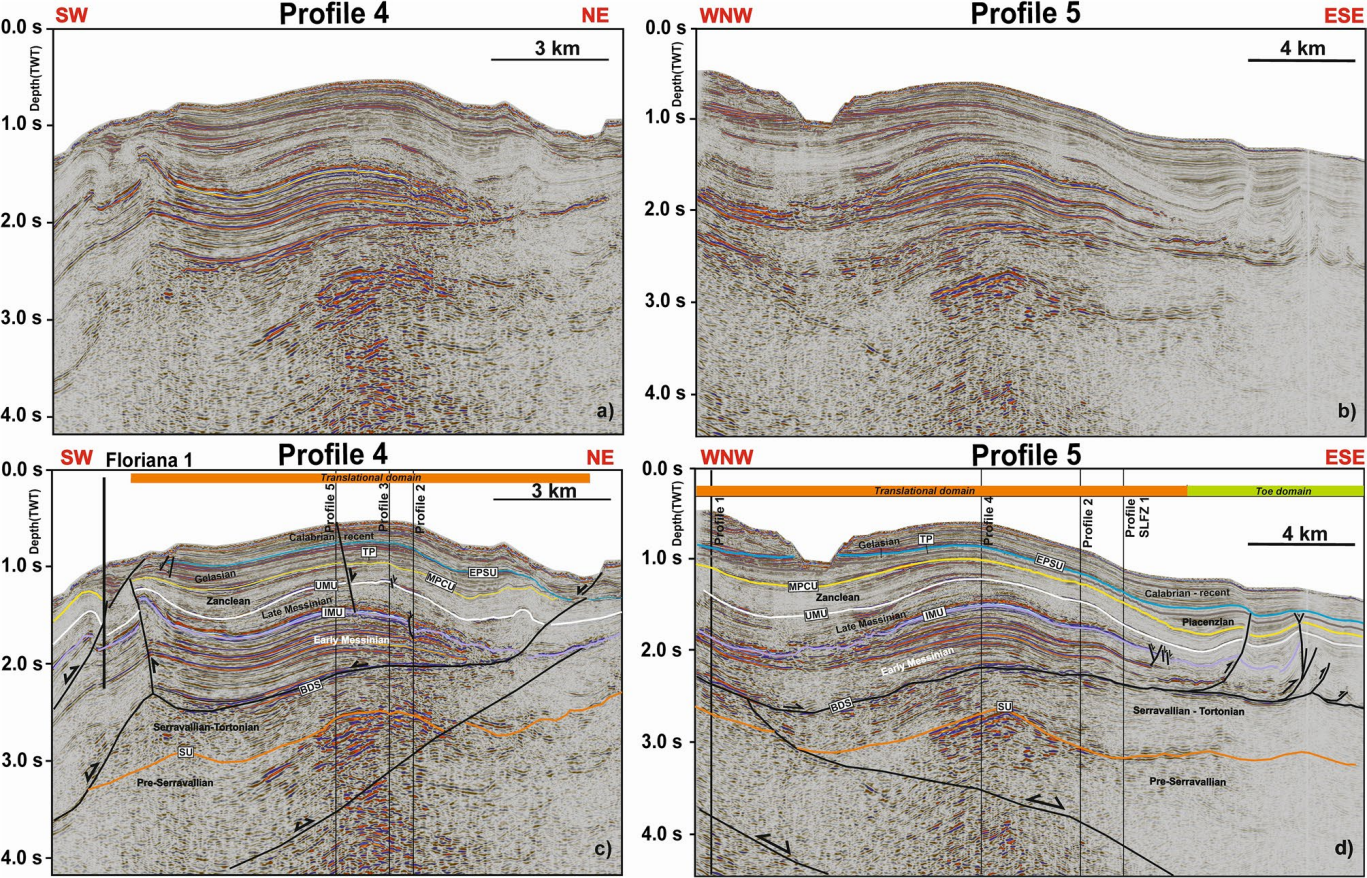
(**a**) Uninterpreted seismic Profile 4 (**b**) uninterpreted seismic Profile 5; (**c**) interpreted SW-NE-oriented seismic Profile 4 imaging the translational domain of the gravitational complex recognized in the Gulf of Squillace. Black-colored segments indicate fault lineaments. (**d**) WNW-ESE-oriented seismic Profile 5 showing the translational domain and the toe region of the *Squillace Complex*. Black-colored segments indicate fault lineaments. Seismic data were provided by ENI Natural Resources (see Fig. 1 for their location).

**Figure 8:**
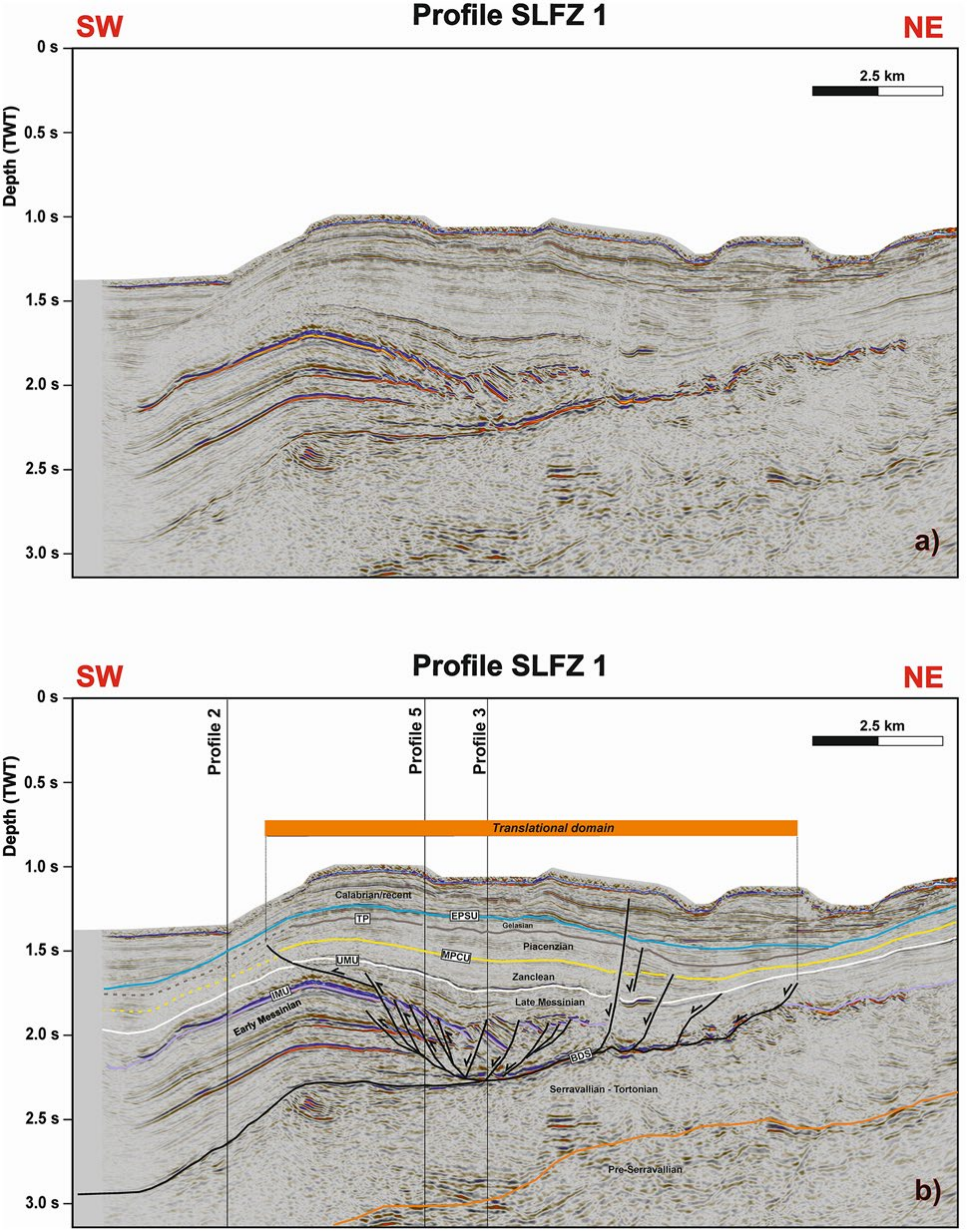
(**a**) Uninterpreted and (**b**) interpreted seismic Profile SLFZ 1. Here, the translational domain of the gravitational complex terminates against an antiform, which is inferred as the offshore prolongation of the Soverato-Lamezia Fault Zone towards the SW. See Fig. 1 for location.

The translational domain is roughly 5 km long and strata are detached above a high-amplitude zone coincident with the base of the gypsum- and anhydrite-rich Messinian sequence, as constrained by wells Lola 1 and Floriana 1 (Figs. 3c,d, 4b, 6). The basal detachment is undulated and forms local ramps that locally erode the older, mud-dominated Ponda Formation (Tortonian) (Fig. 3d). It is overlain by rotated and translated blocks, which retained their internal coherence and continuity (Fig. 3d). In addition, Profile 4 in Fig. 7a and c—a section roughly oriented in a direction parallel to the NE-striking gravity-driven extensional structures—reveals lateral scarps that clearly delimit the translational domain of the *Squillace Complex*. These lateral scarps form linear, continuous features that separate an undeformed region to the NE, and an E-W-oriented flower structure to the SW, from the *Squillace Complex* (Fig. 6). Bathymetric data reveal the lateral scarp to the north as corresponding to the northern flank of a NE- to N-striking high, or spur, inferred to be the seafloor expression of the *Squillace Complex* (Fig. 1). To the S, the lateral scarp is buried under lower Pleistocene (Gelasian) to Holocene strata, as shown in Fig. 3d. On the upper continental slope, this lateral scarp strikes to the NW, changing basinward to a WNW trend, a character suggesting a gross transport direction of the complex towards the ESE (Fig. 1). The lateral scarp is perpendicular to the headwall scarp in the areas where both are observed, and mapped, in seismic data (Figs. 1, 7c and 8b). It is worth noting that this lateral scarp terminates against a contractional feature that is part of the E-W-oriented negative flower structure located in the S sector of the Gulf of Squillace (Figs. 1, 6, 7c and 8a,b). The latter structure is inferred to be the offshore prolongation of the Soverato-Lamezia Fault Zone towards the SW (Fig. 1), where growth strata are observed near the top of the Messinian Unit, along its right-hand fault branch (Fig. 6).

### Toe domain

The toe domain of the *Squillace Complex* is approximately 20 km long and, based on an average p-wave velocity of 2000 m/s, comprises 1 s TWT (approximately 1000 m) high thrusts and associated anticlines cutting through the basal detachment (Figs. 4b, 7d). Thrusts are curved WNW-dipping faults, dipping ~ 3° to ~ 60°, soling out in Messinian to Gelasian strata (Figs. 4b, 5 and 7b,d). The toe domain is thus dominated by the presence of ESE-verging thrusts and associated anticlines (Figs. 4b and 7d). The span of the interpreted 3D seismic data prevents a complete mapping of the toe domain; however, bathymetric data show this toe domain to extend ~ 20 km seawards as a N-striking morphological high (Fig. 1). Such a long toe domain correlates with the displacement observed along its headwall and translation domains. In addition, bathymetric data reveal distinctive slope-parallel sediment undulations, with lengths up to 12 km and a spacing of 0.6–1.4 km. Undulations are up to 100 m tall and asymmetric in their profiles, with steeper upslope-facing flanks^27^.

## Discussion

### Recognition of a new, modern phase of slope instability at the Gulf of Squillace

**Figure 9:**
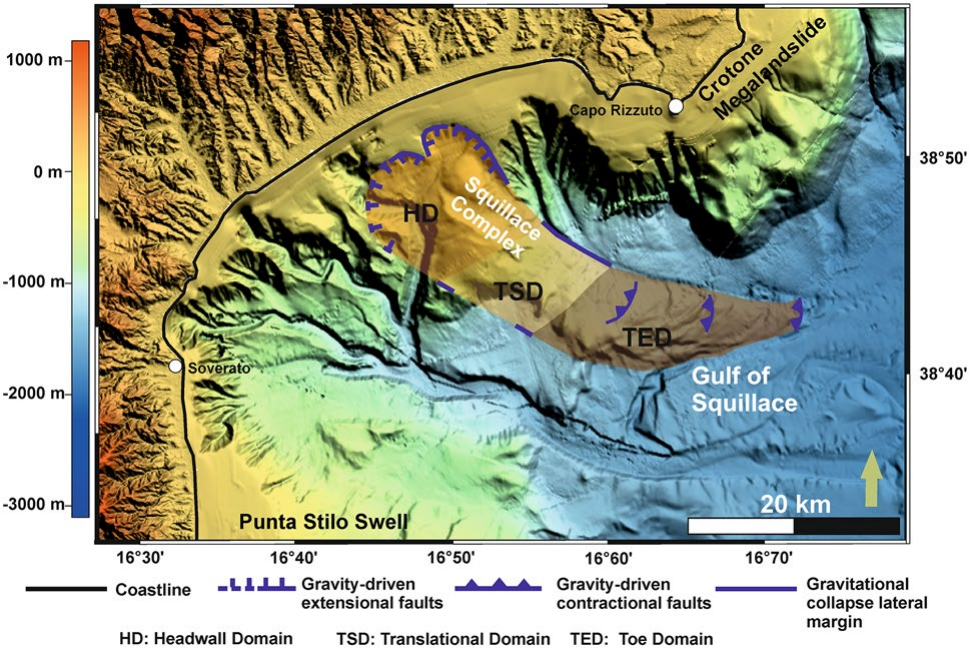
Multibeam bathymetric data highlighting the scale of the *Squillace Complex* within the Gulf of Squillace. The Headwall (HD), translational (TSD) and toe (TED) domains of the complex are mapped in detail. See captions Fig. 1B in order to see where the DTM map was obtained from.

In the Gulf of Squillace, evidence of large-scale gravitational collapse of the continental slope is demonstrated at a distance ~ 1.5 km offshore from the modern coastline—its geometry is akin to gravitational complexes observed on passive continental margins such as SE Brazil^4^, Equatorial Brazil^31^ and around the Gulf of Mexico^32^. In the study area, the *Squillace Complex* reveals a SE-ward movement that changes to the E towards the deeper parts of the margin (Figs. 1 and 9). The complex is ~ 12 km wide and ~ 50 km long, spanning the edge of the continental shelf, the upper and lower continental slope, and thus remobilizing a 600 km^2^ wide area (Figs. 1 and 9). In this work, the full extension of the *Squillace Complex* was inferred in seismic data, allowing for the identification of a basal detachment surface connecting the extensional headwall region to the complex’s contractional toe region.

Based on the identification and dating of growth strata in the *Squillace Complex*, the main phase of gravity sliding started in the Zanclean (early Pliocene) and continued until the Gelasian (early Pleistocene), following an initial SE gross general direction of transport, as evidenced by the NE-SW orientation of the headwall scarps (Figs. 1 and 9). Gravitational gliding slowed down during the Calabrian (middle Pleistocene), as documented by the presence of minor growth strata in Calabrian to Holocene strata in Profiles 3 (Fig. 4), while near-parallel reflectors are observed in correlative strata when interpreting Profiles 1 and 2 (Fig. 3). Movement in the *Squillace Complex* continued toward the present day, as Holocene strata are offset by extensional faults that propagated to the modern seafloor (Fig. 3d). Toward the lower continental slope, a change in the complex’s direction of movement is recognized in both bathymetric and seismic data (Figs. 1, 3 and 9). This may have resulted from the presence of an E-W-oriented flower structure at depth, which forced the complex to move toward the ESE. Such a control is documented on the seismic profiles in Figs. 6, 7c and 8b, where the *Squillace Complex* ends against a transpressional structure (Figs. 6, 7c and 8b). This structure was active during the Late Messinian, as documented by the presence of syntectonic growth strata in the upper part of Messinian strata, and by the formation of a synchronous anticline on its northern flank (Fig. 6).

The Zanclean onset of slope instability followed Late Messinian contractional/transpressional tectonics, which was able to increase local slope gradient^33^. At regional scale, the Late Messinian tectonic phase correlates with incipient collision and temporary coupling of the NE sector of the Calabrian Arc with the Apulian margin, occurred during convergence of western Greece with the Apulian platform^33^. Slope instability was also promoted by a similar contractional/transpressional tectonic event near the Zanclean-Piacenzian boundary. This tectonic event was linked to a temporary pause of the Calabrian Arc migration, due to convergence of the Calabrian accretionary system with continental crust on the Apulian margin^34–39^. At this same time ocean spreading was interrupted, or slowed down, in the Tyrrhenyan back-arc basin^19,40^. In the study area, Late Messinian and Zanclean-Piacenzian contractional/transpresional tectonics resulted in generalized uplift, and associated slope oversteepening, triggering gravitational sliding. In contrast, the phase of slowdown recorded in the Calabrian (middle Pleistocene) can be correlated with important extension affecting the study area, occurring concomitantly with the spreading of the Tyrrhenian Sea and SE migration of the Calabrian Arc^19^. Gravitational collapse of the continental slope during the Holocene was likely promoted by rapid regional uplift of the study area—occurring since the Middle Pleistocene at a rate of 1 mm/year—once again increasing the slope gradient towards the Ionian Sea^41,42^. Middle Pleistocene-Holocene tectonic uplift has been related to: (a) isostatic rebound that followed the breaking of the subducted Ionian oceanic slab^42–44^, and (b) convective removal of the deeper parts of Calabrian Arc and their subsequent delamination from the subducting plate^45^.

### Triggers of slope instability in Southern Italy

Gravitational complexes require the long-lasting presence of an efficient overpressured basal detachment, commonly shaly, acting as a rheologically weak zone^3^. Fluid seepage in the Gulf of Squillace has occurred since the Pliocene^30,39,46^, perfectly matching the onset of slope instability in the study area. A key postulate in this work is that ongoing fluid flow reduces the strength (and friction) of the basal detachment. In fact, our data shows a predominant contact between anhydrite/gypsum-dominated intervals and shaly detachment layers at well Lola 1, located upslope from the *Squillace Complex* headwall region (Fig. 5).

As for the rate of the movement of the *Squillace Complex*, assuming a minimum displacement of ~ 3.6 km observed in the headwall region (Fig. 3d), and taking into account that the complex was active between ca. 4 Ma (Zanclean) and 2.1 Ma (Gelasian), the failed strata moved towards the sea at a rate of ~ 1.9 mm/year. Conversely, considering a maximum offset along the seafloor of ~ 200 m (Fig. 3d) and the slower movement recorded since the Calabrian (middle Pleistocene), gravitational collapse followed a moderate rate of 0.1 mm/year after 1.8 Ma. We stress that the physiography of the study area during the emplacement of the *Squillace Complex* was similar to the present day’s. In addition, its emplacement led to the formation of a clear limit between the upper and lower continental slopes in the study area, as they both follow the trend of the *Squillace Complex* (Figs. 3b, 4).

In spite of the low rate of movement recorded at present in the *Squillace Complex*, the data in this work are helpful to geohazard assessments both in Gulf of Squillace and in similar areas around the world. In fact, just offshore the modern coastline the *Squillace Complex* is seen to be overprinted by a wide canyon system, which may be linked to slope instability and seafloor incision, both important geohazards in the marine environment^27^.

## Data and methods

This study is based on the analysis of a ~ 350 km^2^ 3D seismic volume acquired from the continental shelf to the distal part of the Gulf of Squillace. In addition, a ~ 45 km long 2D profile extending from the onshore sector to the basin depocenter was also interpreted and tied to the 3D seismic volume. Multibeam bathymetric data (10 m of resolution) acquired during the MAGIC project (Marine Geohazard along the Italian Coasts), funded by Italian Department of Civil Protection and OGS, allowed us to constrain the geology and structure of regions with no 3D seismic data. Seismic reflection data and well Lola 1 were provided by ENI Natural Resources. The DTM in this paper was reconstructed by using freely available datasets: U.S. Geological Survey (https://lpdaac.usgs.gov/search/), TinItaly DEM (http://tinitaly.pi.ingv.it/Download_Area2.html), EMODnet Digital Terrain Model (https://www.emodnet.bathymetry.eu/) and Copernicus, the latter of which is the European Union’s earth observation programme (https://land.copernicus.eu/imagery-in-situ/eu-hydro/eu-hydro-river-network-database). Well Floriana 1 was provided by the Ministry of the Economic Development in the framework of the project “Visibility of Petroleum Exploration Data in Italy” (http://www.videpi.com/videpi/videpi.asp) (Table 2).

**Table 2:** Type of data and their sources used in this work.

Data type	Source
Seismic data	ENI natural resources
Lola 1 well	ENI natural resources
Floriana 1 and Liliana 1 wells	Ministry of the economic development http://www.videpi.com/videpi/videpi.asp
Digital terrain model (DTM)	U.S. Geological Survey (https://lpdaac.usgs.gov/search/) TinItaly DEM (http://tinitaly.pi.ingv.it/Download_Area2.html) EMODnet Digital Terrain Model (https://www.emodnet.bathymetry.eu/) Copernicus (https://land.copernicus.eu/imagery-in-situ/eu-hydro/eu-hydro-river-network-database)

**Figure 10:**
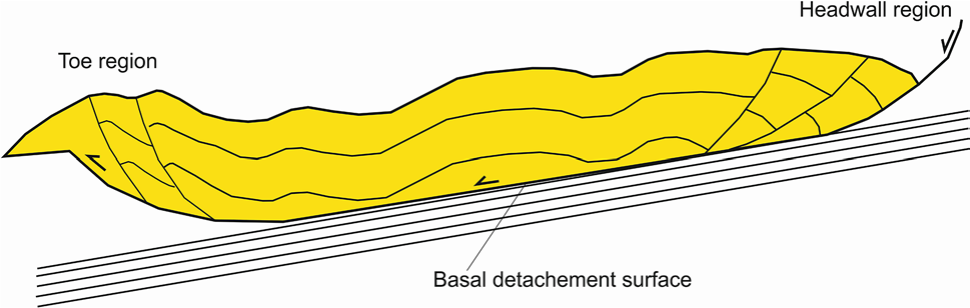
Sketch showing the structure of a typical gravitational complex on a low-angle basal detachment surface, which connects a headwall domain with a toe region (modified from Morley et al.^3^).

The interpretation of seismic and borehole data was completed in the time domain on Schlumberger’s Petrel^©^. Central to the interpretation in this work was the identification of a basal detachment underneath the gravitational complex of interest to this study. This detachment is generally parallel to underlying, undeformed seismic reflectors and overlain by disrupted, chaotic or deformed strata (Fig. 10). Upslope, the identification of the headwall region of gravitational complexes and mass-transport deposits occurs when the basal detachment ramps up and offsets increasingly shallower strata^47–50^. Downslope, the toe region is interpreted near where compressional structures sole out in the basal detachment surface^51^ (Fig. 10).

In order to reconstruct the evolution of slope instability features in the Gulf of Squillace, seismic interpretation was completed based on the recognition of key seismic horizons, and truncation, *downlap* and *onlap* geometries in strata.

## Data Availability

All data generated or analysed during this study are included in this published article.
